# Impact of Asthma on Educational Attainment in a Socioeconomically Deprived Population: A Study Linking Health, Education and Social Care Datasets

**DOI:** 10.1371/journal.pone.0043977

**Published:** 2012-11-14

**Authors:** Pat Sturdy, Stephen Bremner, Gill Harper, Les Mayhew, Sandra Eldridge, John Eversley, Aziz Sheikh, Susan Hunter, Kambiz Boomla, Gene Feder, Keith Prescott, Chris Griffiths

**Affiliations:** 1 Centre for Primary Care and Public Health, Blizard Institute, Barts and The London Medical School, London, United Kingdom; 2 Faculty of Actuarial Science and Statistics, Cass Business School, City University, London, United Kingdom; 3 Department of Public Health Primary Care and Food Policy, Community and Health Sciences, City University, London, United Kingdom; 4 Centre for Population Health Sciences: GP Section, University of Edinburgh, Edinburgh, United Kingdom; 5 Academic Unit of Primary Health Care, University of Bristol, Bristol, United Kingdom; Wayne State University, United States of America

## Abstract

**Background:**

Asthma has the potential to adversely affect children's school examination performance, and hence longer term life chances. Asthma morbidity is especially high amongst UK ethnic minority children and those experiencing social adversity, populations which also have poor educational outcomes. We tested the hypothesis that asthma adversely affects performance in national school examinations in a large cohort from an area of ethnic diversity and social deprivation.

**Methods and Findings:**

With a novel method (using patient and address-matching algorithms) we linked administrative and clinical data for 2002–2005 for children in east London aged 5–14 years to contemporaneous education and social care datasets. We modelled children's performance in school examinations in relation to socio-demographic and clinical variables.

The dataset captured examination performance for 12,136 children who sat at least one national examination at Key Stages 1–3. For illustration, estimates are presented as percentage changes in Key Stage 2 results. Having asthma was associated with a 1.1% increase in examination scores (95%CI 0.4 to 1.7)%,p = 0.02. Worse scores were associated with Bangladeshi ethnicity −1.3%(−2.5 to −0.1)%,p = 0.03; special educational need −14.6%(−15.7 to −13.5)%,p = 0.02; mental health problems −2.5%(−4.1 to −0.9)%,p = 0.003, and social adversity: living in a smoking household −1.2(−1.7 to −0.6)%,p<0.001; living in social housing −0.8%(−1.3 to −0.2)% p = 0.01, and entitlement to free school meals −0.8%(−1.5 to −0.1)%,p<0.001.

**Conclusions:**

Social adversity and ethnicity, but not asthma, are associated with poorer performance in national school examinations. Policies to improve educational attainment in socially deprived areas should focus on these factors.

## Introduction

Asthma is the commonest long-term disorder affecting children in the UK and most economically-developed countries. [1] Health status and education of children are closely linked. [2] In a recent review on asthma in children [3], no mention was made of asthma's impact on educational performance, an important omission given the increasing recognition for a move to assessing impact of long-term conditions on patient/parental-centred outcomes. There is debate about the possible effect of asthma on children's educational performance, with studies producing conflicting results, some finding an adverse effect [4], [5], [6], some no effect [7], [8], [9], [10], and others a beneficial effect. [11], [12] Taras [13] proposed asthma was related to poor exam performance and called for evaluations focusing on populations at increased risk.

Populations at particular risk are children of low socioeconomic status and children of south Asian and Black ethnic minority origin: both experience increased asthma morbidity and poor educational attainment. [14], [15] However, no study has examined the impact of asthma on school examination performance in children from large ethnically diverse socio-economically deprived populations. Results of such a study would help guide policy by identifying and then targeting potentially modifiable factors that relate to poor educational attainment.

We therefore tested the hypothesis that asthma worsens educational attainment in children from socio-economically deprived, multiethnic populations. Tower Hamlets, in east London, is the UK's third most deprived borough. [16] Its population is largely White or Bangladeshi, providing an ideal setting for this study. The study received Local Research Ethics Committee approval.

## Methods

### Study participants

Fifteen general practices in Tower Hamlets were approached and 14 participated in this cross-sectional study covering 1^st^ July 2001 to 30^th^ June 2005. These practices provided care for almost 50% of the borough's children. Inclusion criteria for practices were use of the EMIS computer system (one of the main software suppliers to UK general practices), [17] and a list of over 5,000 patients (to maximise our dataset). We undertook MIQUEST (Morbidity Information QUery and Export SynTax) searches on all patients aged under 20 on 30 June 2005. For confidentiality, clinical data were collected separately from administrative data. We included all who had sat at least one national Key Stage 1–3 attainment test between 2002 and 2005 (table 1), identified on the Annual School Census (ASC), which includes all children attending Tower Hamlets state schools. National Key Stage tests assess children in England, Wales and Northern Ireland against the content of the National Curriculum, and so provide a standardised comparison of academic performance. Details of the children included are given in table 2.

**Table 1:** Key stage tests for the UK's National Curriculum.

Key stage	School year	Approx pupil age	Topics assessed	maximum mark attainable
Key stage 1	Year 2	7 years	English, maths, science	30
Key stage 2	year 6	11 years	English, maths, science	100
key stage 3	Year 9	14 years	English, maths, science	150

**Table 2:** Characteristics of 12,136 pupils that sat Key Stage tests in 2002 to 2005.

School attainment data	variable	n	%/SD/range
number of pupils with results only for	KS 1	4074	(33.6%)
	KS 2	3720	(30.7%)
	KS 3	3360	(27.7%)
number of pupils that took two KS tests	KS 1 & 2	16	(0.1%)
	KS 2 & 3	966	(8.0%)
attainment (English): mean (SD)	KS 1	13.7	(4.0)
	KS 2	52.5	(18.1)
	KS 3	34.9	(17.8)
attainment (Maths): mean (SD)	KS 1	15.2	(4.0)
	KS 2	60.7	(21.9)
	KS 3	66.9	(23.8)
attainment (overall): mean (SD)	KS 1	14.2	(3.7)
	KS 2	56.0	(16.0)
	KS 3	60.8	(18.7)
special educational needs (at KS)	KS 1	807	(19.7)
	KS 2	1,117	(23.8)
	KS 3	971	(22.4)
Clinical data			
asthma diagnosis (ever)		2,206	(18.2%)
asthma treatment	inactive	1,125	(9.3%)
	active	946	(7.8%)
bronchodilators: 50th (10th–90th) centiles	3 months†	1.8	(1.3 to 3.6)
	12 months‡	3.4	(1.4 to 8.0)
BTS Step in 2005	1	767	(6.7%)
	2	450	(3.9%)
	3+	153	(1.3%)
comorbidites	any atopic disease	3,483	(28.7%)
	allergic rhinitis	1,266	(10.4%)
	eczema	2,592	(21.4%)
	mental health problems	521	(4.3%)
	diabetes	19	(0.2%)
smoking household	yes	6,679	(55.0%)
	1 smoker	4,474	(67.0%)
	2 smokers	1,572	(23.5%)
	3+ smokers	633	(9.5%)
Socio demographic data			
Sex	males:	6,091	(50.2%)
ethnicity	White/NA	3,216	(26.5%)
	Bangladeshi	7,126	(58.7%)
	other	1,794	(14.8%)
Council Tax band*	A: ≤£40 k	29	(0.3%)
	B: >£40 k to ≤£52 k	2,533	(21.6%)
	C: >£52 k to ≤£68 k	5,856	(49.9%)
	D: >£68 k to ≤£88 k	1,392	(11.9%)
	E: >£88 k to ≤£120 k	1,314	(11.2%)
	F: >£120 k to ≤£160 k	544	(4.6%)
	G: >£160 k to ≤£320 k	67	(0.6%)
	H: >£320 k	0	(0%)
in receipt of benefits		8,646	(71.2%)
living in social housing		8,188	(67.5%)
one adult household		1,370	(11.3%)
free school meals		7,607	(62.7%)

KS 1 sat aged 7, KS 2 sat aged 11, KS 3 sat aged 14.

SD = standard deviation.

range = 10th to 90th percentile.

BTS = British Thoracic Society.

active asthma = bronchodilator in period 12 months before Key Stage test.

inactive asthma = no bronchodilator in period 12 months before Key Stage test.

† = in period 3 m prior to KS test.

‡ = in period 12 m prior to KS test.

* Council Tax band based on property valuation on 1st April 1991.

NA = not available.

### Outcome variable (table 2)

The outcome variable was level of individual attainment at Key Stages 1, 2, and 3. Children who sat more than one Key Stage test contributed more than one observation to the data. For Key Stage 1 the mean score of reading, writing and mathematics and for Key Stages 2 and 3 the mean score of English, mathematics and science was calculated for each pupil for 2002 to 2005.

### Predictor variables (table 2)

Our primary predictor was asthma status coded in three groups: 1) no diagnosis of asthma; 2) diagnosis of asthma with one or more bronchodilator prescriptions in the relevant year (‘*active asthma*’), and 3) diagnosis of asthma but no bronchodilator prescription in the year before July 1^st^ of the year in which the Key Stage test was sat (‘*inactive asthma*). Data on asthma status were obtained from practice records. We used the H33 Read code allocated by the practice to identify children with asthma. In two practices where coding of asthma was poor, any child receiving repeat prescriptions of long acting bronchodilators and/or inhaled corticosteroids was allocated an asthma code, as were those with six or more prescriptions of short acting bronchodilators over a four year period.

*Asthma severity* was assessed by using British Thoracic Society (BTS) medication step as a proxy. We assigned each child a BTS step by examining their prescriptions in the final year of the study. [18]

*Asthma control* was assessed by estimating numbers of short-acting bronchodilator devices prescribed per child per year as a proxy. Whilst MIQUEST searches do not provide the actual number of inhalers prescribed on a given prescription, we examined the individual prescriptions of 50 randomly selected asthmatic children in each practice to find the average number of short-acting bronchodilator inhalers prescribed per prescription and thus estimated the number of devices prescribed per child.

*Ethnicity* was obtained from ASC data and was categorised into three groups: 1) White or ‘ethnicity not recorded’, 2) Bangladeshi, and 3) ‘other ethnicity’. Coding of Tower Hamlets minority ethnic group schoolchildren is almost 100% complete; we assumed the few children with ‘ethnicity not recorded’ (1.6% of total) were likely to be White, and checked this in sensitivity analyses.

*Social adversity* was measured using socio-economic information at the household level from the Housing Department of the London Borough of Tower Hamlets: the Council Tax band of the child's residence (defined by value of dwelling on 1^st^ April 1991), whether it was of ‘social housing’ tenure, if anyone at that address was in receipt of Council Tax or Housing Benefit and if it was a ‘one adult’ household. The ASC provided information on eligibility for free school meals, special educational needs, exclusion from school, name and type of school attended (mixed or single sex). Practice records provided data on presence of a smoker in household, number of smokers, and possible co-morbidities including allergic rhinitis, eczema, glue ear, diabetes, chronic tonsillitis and mental health problems.

### Data linkage

We extracted an ‘Administrative File’ from practice computers consisting of names and addresses of children and a discrete Patient ID number derived from a randomly-generated practice number concatenated with the patient's EMIS number. A single merged Administrative File from all the participating practices formed the foundation of the Master Dataset (in Microsoft Access) onto which socioeconomic, educational, and finally clinical data were merged. The procedure by which we linked data was as follows:

#### i) Assigning socio and demographic data using matching by address

We assigned each address on the Master Dataset a Unique Property Reference Number (UPRN) from the Local Land and Property Gazetteer. This overcame the problem of differently formatted versions of the same address in different datasets. Using the UPRN, we then linked socio-economic information at the household level to the Master Dataset, including data from Council Tax Banding and Council Tax Benefit datasets. Using address, we also linked data from general practices on which households had smokers.

#### ii) Assigning school attainment data

We ascribed each pupil a unique pupil number (UPN, derived from the Annual School Census) and added these to the Master Dataset using matching by name, date of birth, UPRN, and postcode. This was an iterative process requiring detailed checks at each stage. Matching difficulties arose, for example, where first names were shortened or incorrectly spelt, surnames given with middle names, dates of birth incorrectly entered or postcodes changed. Unmatched records were subjected to successive relaxations of the criteria and subsequent resultant matches were scrutinised for errors. Once the matching process was complete, we identified possible twins using duplicate surnames and dates of birth. We checked these records manually to ensure we had assigned the correct ASC data to the correct twin.

All children on the Master Dataset were found their corresponding match on the 2002 to 2005 Annual School Census data – none remained unmatched. If a child did not exist on Census data, this indicated they did not attend a Tower Hamlets state school during this period, and no pupil or Key Stage data could be assigned to them. They were therefore excluded from the analysis.

#### iii) Pseudonymising the database for confidentiality

Until this point the Master Dataset contained patient identifying administrative details. To pseudonymise the dataset we then removed these to leave only the discrete Patient ID number, age and sex.

#### iv) Assigning general practice clinical and prescribing data

Our clinical and prescribing data from MIQUEST searches included the discrete Patient ID number but no other patient details. Using this discrete ID number we then matched and attached the clinical and prescribing data to the pseudo-anonymised database, thus retaining confidentiality.

### Statistical methods

#### Sample size

We anticipated 20,000 children would have analysable data, 20% of whom would have asthma. Allowing for clustering of attainment by school, and assuming 100 children per school and an intra-cluster correlation coefficient (ICC) (for the difference in attainment between children with and without asthma) of 0.05, gives, working back, a simple sample size of 3,361 children using the formula N_simple_ = N_cluster_/(1+(100-1)*ICC). With 80% power at the 5% significance level, this would allow us to detect a difference in standardised overall attainment of 0.12 standard deviations between 672 children with asthma and 2689 children without.

#### Analysis

Analysis was carried out in Stata 10.1 [19] and involved regression of attainment on the predictor variables and potential confounders. To maximise power we combined all years and all Key Stages. Attainment could be affected by whether a child is old or young for their year. We therefore included a variable which reflected this: age was standardised within each academic year to have a mean of 0 and SD of 1. Assuming that Key Stage tests varied in difficulty between years, attainment was also standardised within each academic year. Standardised attainment was regressed, in a linear model, on standardised age, and several socio-demographic and clinical variables pertaining to asthma and co-morbid conditions. We used data reduction techniques to decide on the variables to consider for this multiple regression, including removing binary variables which would not be discriminatory, and removing socio-demographic variables for which P>0.1 in bivariate regression models. The final selection of variables was: asthma control in the 12 months prior to the Key Stage test, ethnicity, standardised age, sex, living in a smoking household, living in a property in Council Tax bands A, B or C, living in social housing, in receipt of Council Tax/housing benefit, in receipt of free school meals, having special educational needs, diagnoses of allergic rhinitis, eczema, and mental health problems. In primary analysis asthma control was noted in the 12 months prior to Key Stage tests. We fitted a multiple linear regression model with robust standard errors using White's sandwich estimator, implemented by specifying school as the clustering variable. [20] This overcame the underestimation of the standard errors due to the tendency for children within schools to be more alike than children between schools (intra-cluster correlation).

The percentage change in test scores for a unit change in a regression coefficient are given in appendix column 2. These were obtained using the following multiplication factors (KS1: 12.3, KS2: 16, KS3: 12.5), which were calculated from SD(KS test)×100/max possible score for KS test).

We conducted six separate sensitivity analyses to investigate the effect of varying assumptions on our model estimates (Table S1). These were: defining inactive asthma as having no bronchodilator for the last three (instead of twelve) months, excluding a more recent Key Stage test result where a pupil had sat two tests to remove the interdependence of test results for the same child, using three different ways of grouping ethnicity and not imputing an asthma diagnosis in 79 children registered at two practices.

Finally, for the subset of children for whom BTS step could be ascertained in the final year of the study, we fitted the same model as in the primary analysis except that we first stratified our data by active or inactive asthma, and included an indicator variable for BTS Step 2, 3, 4 or 5 versus 1 and a variable giving the estimated average number of bronchodilator prescriptions in the 12 months prior to the Key Stage test.

Barking and Havering Local Research Ethics Committee gave full ethical approval on 6th October 2005 for the study methodology (REC reference number: 05/Q0602/83). The Research Ethics Committee did not require written consent to be given by the patients next of kin, carers or guardians on the behalf of the minors/children for their information to be stored in the hospital database and used for research. A STROBE checklist is provided as a supplement.

## Results

Our search identified 30,841 children aged 0–19 years old registered with the 14 study practices. Of these, 20,683, were identified on the Tower Hamlets Schools Census Data, ie they had attended a Tower Hamlets school during the study period, the remainder (10,158) being too young to attend school. Of the 20,683, we retained those who were aged 5–14 years old and had sat at least one Key Stage test. This produced a database that contained observations on 12,136 children, from 97 schools, who had sat at least one of Key Stage tests 1, 2 or 3 (Table 2). 2,206 (18.2%) children had a diagnosis of asthma. The majority of children (1,370) that had BTS step recorded had mild asthma (767 children) (step 1). A third were at step 2 and the remainder at step 3 or above. Allergic rhinitis was recorded in 10.4%, eczema in 21.4% and atopy in 28.7%. Mental health problems (largely behavioural and developmental) were diagnosed in 4.3% of children.

58.7% of children were Bangladeshi, with 26.5% White or ‘ethnicity not recorded’ (1.6% of all children) and 14.8% ‘other ethnicity’. Most children lived in socioeconomically deprived circumstances. Council Tax bands A, B or C (the lowest valued dwellings) were recorded against 71.8% of addresses. Almost three quarters of children were from families receiving housing and/or Council Tax benefit. Just over two thirds of children lived in social housing and 11.3% were from households with one resident adult. A majority (55.0%) of children lived in smoking households.

The estimated median number of inhaler devices prescribed in the three month period pre Key Stage test was 1.8 (10^th^–90^th^% spread 1.3–3.6). In the 12 months prior to the Key Stage test, the median number of devices prescribed was 3.4 (1.4–8.0). 21.5% of children with asthma had a prescription for a bronchodilator in the three months prior (recent asthma control) to the school examination, rising to 45.1% in the period 12 months prior to their examination.

### Primary analysis

Analysis of attainment for Key Stages 1, 2 and 3 combined is presented in table 3. For ease of interpretation, we have presented these results in the abstract as percentage changes in examination scores for Key Stage 2 – the most commonly sat examination in the dataset. Regression coefficients are presented here (for percentage changes in scores for all Key Stages see appendix table 3). As the outcome is standardised overall attainment, the interpretation of a particular regression coefficient is, for all other variables fixed, the proportion of a standard deviation change in attainment given a one unit change in the predictor variable of interest. As age was also standardised, a one unit change in age is equivalent to a one SD change in age (approx 2.8 years). 22% of the variability in standardised overall attainment was explained by the model.

**Table 3:** Coefficients, 95% confidence intervals and P-values for the effect of socio demographic and clinical variables on standardised attainment scores in Key Stage tests 1, 2 and 3, from multiple regression model allowing for clustering.

			simple regression							multiple regression*				
independent variable	β		95% CI				p-value	β		95% CI				p-value
† inactive asthma vs. no asthma	0.047	(	−0.011	to	0.105	)	0.11	0.023	(	−0.025	to	0.071	)	0.35
‡ active asthma vs. no asthma	0.053	(	−0.007	to	0.112	)	0.08	0.066	(	0.013	to	0.119	)	0.02
Bangladeshi vs. White	−0.070	(	−0.155	to	0.016	)	0.11	−0.082	(	−0.157	to	−0.007	)	0.03
other ethnicity vs. white	0.052	(	−0.021	to	0.125	)	0.16	0.008	(	−0.054	to	0.071	)	0.79
standardised age	0.086	(	0.064	to	0.108	)	<0.001	0.053	(	0.034	to	0.071	)	<0.001
girls vs. boys	0.108	(	0.067	to	0.149	)	<0.001	0.003	(	−0.042	to	0.048	)	0.89
smoking household (Yes vs. No)	−0.132	(	−0.172	to	−0.093	)	<0.001	−0.072	(	−0.107	to	−0.037	)	<0.001
Council Tax band (A–C vs. D–H)	−0.099	(	−0.146	to	−0.052	)	<0.001	−0.059	(	−0.094	to	−0.025	)	0.001
living in social housing (Yes vs. No)	−0.144	(	−0.185	to	−0.104	)	<0.001	−0.047	(	−0.082	to	−0.011	)	0.01
in receipt of benefits (Yes vs. No)	−0.236	(	−0.293	to	−0.179	)	<0.001	−0.115	(	−0.163	to	−0.066	)	<0.001
free school meals (Yes vs. No)	−0.192	(	−0.242	to	−0.142	)	<0.001	−0.051	(	−0.093	to	−0.008	)	<0.001
special educational needs (Yes vs. No)	−0.943	(	−1.013	to	−0.872	)	<0.001	−0.912	(	−0.981	to	−0.842	)	0.02
allergic rhinitis diagnosis (Yes vs. No)	0.056	(	0.005	to	0.106	)	0.03	0.022	(	−0.022	to	0.067	)	0.32
eczema diagnosis (Yes vs. No)	0.041	(	−0.003	to	0.085	)	0.07	0.018	(	−0.025	to	0.061	)	0.41
mental health problems (Yes vs. No)	−0.281	(	−0.407	to	−0.155	)	<0.001	−0.154	(	−0.256	to	−0.052	)	0.003
constant term								0.533	(	0.421	to	0.645	)	<0.001

β = regression coefficient.

† No prescription for a bronchodilator in the 12 months before the Key Stage test, but asthma diagnosis ever.

‡ At least one prescription for a bronchodilator in the 12 months before the Key Stage test and asthma diagnosis ever.

* model includes all variables listed.

The intra-school correlation coefficient in the multiple regression model is 0.06, 95% CI (0.04 to 0.08).

#### Asthma status

A weak positive association was found between overall school examination attainment and having active asthma (asthma treated with a bronchodilator during the past 12 months): β = 0.066 (95% CI 0.013 to 0.119). No association was found for inactive asthma: β = 0.023 (95% CI −0.025 to 0.071). For these groups (active and inactive asthma) combined, a weak positive association was found: β = 0.04 95% CI (0.00 to 0.08).

#### Ethnicity

Bangladeshi children did significantly worse in the Key Stage tests than White children β = −0.082 (95% CI −0.157 to −0.007), though there was no evidence of a difference between children of ‘other’ ethnicity and White children: β = 0.008 (95% CI −0.054 to 0.071).

#### Social adversity

Statistically significant associations were observed between attainment and several socio-demographic variables. Children from smoking households: β = −0.072 (95% CI −0.107 to −0.037), living in social housing: β = −0.047 (95% CI −0.082 to −0.011), in receipt of housing/Council Tax benefit: β = −0.059 (95% CI−0.094 to −0.025), and receiving free school meals: β = −0.051 (95% CI −0.093 to −0.008) did significantly worse in the tests than children from less deprived households. Children identified as having special educational needs: β = −0.912 (95% CI −0.981 to −0.842) or with mental health problems: β = −0.154 (95% CI −0.256 to −0.052) also performed significantly worse. Girls did not score significantly higher than boys: β = 0.003 (95% CI −0.042 to 0.048). Children that were older for their year did better than younger children: β = 0.053 (95% CI 0.034 to 0.071). There was no association between eczema: β = −0.018 (95% CI −0.025 to 0.061) or allergic rhinitis: β = −0.022 (95% CI −0.022 to 0.067) and overall attainment.

#### Asthma severity and attainment

We examined whether children with more severe asthma might have poorer examination scores. Table 4 gives the adjusted effects of asthma severity (BTS step) and other predictor variables on standardised attainment from two separate multiple regression models (active asthma: 882 children, inactive asthma: 462 children). We found no association between BTS step and attainment in children. Furthermore, bronchodilator use in those with active asthma was not associated with attainment in the Key Stage tests.

**Table 4:** Coefficients and 95% confidence intervals for the adjusted effect of asthma severity (BTS step) and other predictor variables on standardised attainment, from two separate multiple regression models (active asthma: 882 children, inactive asthma: 462 children).

	asthma	β	95% CI for β		P-value
BTS Step 2,3,4 or 5 vs. 1	inactive	−0.098	−0.259	0.062	0.23
	active	−0.003	−0.097	0.091	0.95
Bronchodilator use (last 12 months)	inactive	NA	NA	NA	NA
	active	0.000	−0.017	0.017	0.98
Bangladeshi vs. White	inactive	−0.110	−0.291	0.071	0.23
	active	0.044	−0.082	0.169	0.49
Other ethnicity vs. White	inactive	0.022	−0.125	0.169	0.76
	active	0.125	−0.019	0.269	0.09
standardised age	inactive	0.095	0.019	0.171	0.01
	active	0.079	0.022	0.136	0.007
girls vs. boys	inactive	0.057	−0.081	0.194	0.42
	active	−0.086	−0.177	0.006	0.07
smoking household (Yes vs. No)	inactive	−0.127	−0.253	−0.001	0.05
	active	−0.020	−0.136	0.095	0.73
Council Tax band (A–C vs. D–H)	inactive	0.038	−0.091	0.168	0.56
	active	0.052	−0.101	0.205	0.50
living in social housing (Yes vs. No)	inactive	−0.029	−0.206	0.149	0.75
	active	−0.045	−0.195	0.105	0.55
in receipt of benefits	inactive	−0.003	−0.172	0.165	0.97
	active	−0.097	−0.245	0.051	0.20
free school meals (Yes vs. No)	inactive	−0.190	−0.330	−0.049	0.009
	active	−0.076	−0.185	0.043	0.207
special educational needs (Yes v No)	inactive	−0.880	−1.067	−0.689	<0.001
	active	−1.024	−1.210	−0.838	<0.001
allergic rhinitis diagnosis (Yes vs. No)	inactive	−0.038	−0.185	0.108	0.60
	active	−0.004	−0.130	0.122	0.95
eczema diagnosis (Yes vs. No)	inactive	−0.076	−0.228	0.075	0.32
	active	0.063	−0.042	0.168	0.24
mental health problems (Yes vs. No)	inactive	−0.143	−0.562	0.276	0.50
	active	0.197	−0.036	0.429	0.10
constant term	inactive	0.593	0.239	0.947	0.001
	active	0.572	0.337	0.806	<0.001

## Discussion

### Summary

We found no evidence for an adverse effect of asthma or asthma severity on examination performance in this large cross sectional study of children from a highly socio-economically deprived, multiethnic area. There was a very small positive association between attainment and having active asthma in the 12 months prior to the Key Stage test *versus* having no asthma. With respect to asthma these findings are reassuring for parents and teachers alike.

Examination performance was poorer in Bangladeshi children, and those experiencing social adversity (eligibility for free school meals, living in social housing, ‘one parent’ households and households with a smoker), those with mental health problems and special educational needs, suggesting that these should be the foci of policies to improve educational attainment.

### Strengths and weaknesses

Strengths include use of large unbiased datasets in a locality with characteristics ideally suited to testing the study hypothesis: high asthma prevalence, socioeconomic deprivation and ethnic diversity with two groups (White and Bangladeshi) predominating with accurate and near complete coding of ethnicity. Excellent participation by local general practices allowed us to capture data on half the children in the borough sitting school examinations. A sophisticated methodological approach allowed us to link general practice, housing and educational authority databases, enabling us to address a wide range of clinical and socio-demographic factors, and importantly, to explore relationships between clinical factors and social outcomes. We believe this is the first time these disparate data sources have been merged. Our ability to match Unique Property Reference Numbers and Unique Pupil Numbers to general practice administrative data enabled us to identify households of those living in poor socio-economic circumstances, households with smokers, and to identify ethnicity, those receiving free school meals and children with special educational needs, as well as providing examination results. This methodology provides a useful tool for further epidemiological research.

Our finding that aspects of social adversity were associated with poor educational outcomes is in keeping with previous work, see for example Richards, [21] and is a good indication of the validity of our approach and the linked dataset generated. These latter variables are more plausible influences on school exam performance than asthma. Data on absenteeism may have helped to elucidate our findings as school absence in children with asthma has been shown to vary with ethnicity and housing conditions. [22], [23] It was disappointing that some markers of asthma severity including peak flow recording and asthma consultations were poorly recorded in general practice. Relatively few children with very severe asthma meant it is difficult to generalise our findings to that population. Extrapolating BTS step for a single year relied on the assumption that severity for each child was stable over the study duration. We did not use mediating and/or moderating models in our analysis, and it is possible that at some level of social disadvantage having asthma might have an adverse impact. Possible explanations for our finding of a very small positive association between asthma and attainment in the 12 months prior to a Key Stage test are speculative but include a chance effect, a selection effect (for example, asthma occurring in brighter children), or a behavioural effect (children with asthma studying or performing in tests in a different way).

### Comparison with other studies

Previous studies fall into three categories: (i) retrospective studies measuring educational level reached in adults with asthma [4], [5], (ii) case-control studies investigating results of examinations in children with and without asthma, [8], [9] (iii) comparison of examination results of children with asthma and general population data. [11], [12] Two retrospective studies have linked a diagnosis of asthma with lower levels of education [4], [5] and two studies found a positive association with above average scores. [11], [12] Our study is consistent with the majority of other studies in not finding an association between poor attainment and asthma. [8], [9], [10] Our results could reflect well-controlled asthma. [24] One study [6] found that children with severe asthma performed worse in examinations - an association we did not find, even when examining children at BTS step 3.

### Clinical and policy relevance

Our work suggests the important drivers of poor performance relate not to asthma, but to ethnicity, social adversity, mental health problems and special educational needs. These should be the foci of policies to improve educational attainment. Pakistani and Bangladeshi children are amongst the lowest achieving groups in the UK. [25] Reasons for their poorer performance could be related to language difficulties and absenteeism. Two American studies have shown that black children miss more school than white. [26], [27] Social adversity was significantly associated with lower test scores. Biological and psychosocial mechanisms might explain this relationship. [21] While children from lower socio-economic backgrounds experience more morbidity [28] including asthma [29]–[32] this finding was independent of asthma diagnoses and ethnicity. It is plausible that children with special educational need and those with a history of mental health problems fared worse in tests, especially as behavioural difficulties and developmental problems made up a substantial proportion of the latter.

Our classification of asthma severity by medication step might suggest that most children have mild asthma. This interpretation warrants caution. East London has the highest rates of hospital admission for respiratory illness in children in London, much of which is likely to be asthma related. Future work should explore possible under-treatment in this locality.

### Conclusion

Our results provide the first large scale assessment of the relationship between asthma, and educational attainment in an ethnically diverse and socioeconomically deprived population. Our results are reassuring with respect to effects of asthma on educational attainment, but provide reason for policymakers to prioritise social adversity and mental health problems as drivers of poor exam performance. Our data linkage methodology has the potential to be applied in other areas as a means to inform health care policy by quantifying links between demography and clinical and social outcomes.

## Supporting Information

Table S1 Sensitivity analyses.
(DOCX)
