# Supplementary material for: Impact of Asthma on Educational Attainment in a Socioeconomically Deprived Population: A Study Linking Health, Education and Social Care Datasets
Source: PLoS One. 2012 Nov 14;7(11):e43977. doi: 10.1371/journal.pone.0043977 (PMC3498297; doi:10.1371/journal.pone.0043977)
Supplement: Table S1 — Sensitivity analyses. (DOCX) [file pone.0043977.s001.docx]

**Sensitivity analysis**

The appendix table presents regression coefficients and their standard errors for six separate models to assess the sensitivity of the regression coefficients for the asthma control variables to different specifications of ethnic groups, time window defining active asthma (prescription of a bronchodilator within either three or twelve months prior to Key Stage test), having results for one or two Key Stage tests and removing the children for whom a diagnosis of asthma had been imputed from their prescription history. The first column shows the primary model presented in table 2. The estimates are not particularly sensitive to different model specifications and the overall percentage of the variation in attainment explained was 22% in every case. There was no association with short-term control of asthma and overall attainment. To satisfy the linear regression assumption of independent errors, the second test sat by children with results for two Key Stage tests was dropped from the data set (1,002 observations). The regression coefficient comparing attainment in children with treated asthma to children with no asthma diagnosis was 0.069 and significant at the 5% level. The three models specifying different ethnicity groupings (A, B and C) led to very similar conclusions for the association between treated and untreated asthma and overall attainment. In conclusion, we found some evidence of a small (approx 0.07 SDs) positive association between having asthma and at least one bronchodilator prescription in the 12 month period prior to a Key Stage test, and overall attainment in the test. There were 79 children for whom a diagnosis of asthma was imputed on the basis of their prescribing history alone. Analysing them as children without asthma instead made virtually no difference to the results.

Appendix table S1. Sensitivity of effects on standardised attainment to a variety of model specifications. The intra-school correlation coefficient in each of these models is estimated to be 0.06 95% CI (0.04 to 0.08).

Key

β = regression slope, se(β) = standard error of regression slope

12m control = bronchodilator prescription in 12 months prior to Key Stage test

3m control = bronchodilator prescription in 3 months prior to Key Stage test

In the first three (models) columns, ethnicity 2 vs. 1 refers to Bangladeshi vs. White/unknown, and ethnicity 3 vs. 1 refers to ‘other ethnic groups’ vs. White/unknown

In the ethnicity A model, ethnicity 2 vs. 1 refers to Bangladeshi vs. White, and ethnicity 3 vs. 1 refers to ‘other ethnic groups’ vs. White (unknown excluded)

In the ethnicity B model, ethnicity 2 vs. 1 refers to Bangladeshi vs. White, and ethnicity 3 vs. 1 refers to ‘other ethnic groups’/unknown vs. White

In the ethnicity C model, ethnicity 2 vs. 1 refers to Bangladeshi/other vs. White/unknown.

NA = not applicable

No imputed asthma: despite evidence of asthma in the medication history in the absence of a Read code, a diagnosis was not imputed.
